# Single nucleotide polymorphisms (SNPs) in the open reading frame (ORF) of prion protein gene (*PRNP*) in Nigerian livestock species

**DOI:** 10.1186/s12864-024-10070-2

**Published:** 2024-02-14

**Authors:** Adeniyi C. Adeola, Semiu F. Bello, Abdussamad M. Abdussamad, Rahamon A. M. Adedokun, Sunday C. Olaogun, Nasiru Abdullahi, Akanbi I. Mark, Anyebe B. Onoja, Oscar J. Sanke, Godwin F. Mangbon, Jebi Ibrahim, Philip M. Dawuda, Adebowale E. Salako, Samia Kdidi, Mohamed Habib Yahyaoui

**Affiliations:** 1 State Key Laboratory of Genetic Resources and Evolution & Yunnan Laboratory of Molecular Biology of Domestic Animals, Kunming Institute of Zoology, Chinese Academy of Sciences, Kunming, China; 2 Sino-Africa Joint Research Center, Chinese Academy of Sciences, Kunming, China; 3 Department of Animal Genetics, Breeding and Reproduction, College of Animal Science, South China Agricultural University, 510642 Guangzhou, China; 4 Department of Veterinary Physiology and Biochemistry, Faculty of Veterinary Medicine, Bayero University, Kano, Nigeria; 5 Department of Veterinary Medicine, Faculty of Veterinary Medicine, University of Ibadan, Ibadan, Nigeria; 6 Department of Biochemistry, Faculty of Basic Medical Sciences, College of Health Sciences, Bayero University, Kano, Nigeria; 7 Ministry of Agriculture and Rural Development, Secretariat, Ibadan Nigeria; 8 Department of Virology, College of Medicine, University of Ibadan, Ibadan, Nigeria; 9 Taraba State Ministry of Agriculture and Natural Resources, Jalingo, Nigeria; 10 Division of Veterinary Office, Serti, Nigeria; 11 Department of Veterinary Surgery and Theriogenology, College of Veterinary Medicine, University of Agriculture Makurdi, Makurdi, Nigeria; 12 Department of Animal Science, Faculty of Agriculture, National University of Lesotho, Maseru, South Africa; 13 Department of Animal Science, Faculty of Agriculture, University of Ibadan, Ibadan, Nigeria; 14 Livestock and Wildlife Laboratory, Institut des Régions Arides, Université de Gabes, Route El Djorf, Km 22.5, 4119 Medenine, Tunisia

**Keywords:** Scrapie, Susceptibility, Prion protein gene, Polymorphism, Livestock, Nigeria

## Abstract

**Background:**

Prion diseases, also known as transmissible spongiform encephalopathies (TSEs) remain one of the deleterious disorders, which have affected several animal species. Polymorphism of the prion protein (*PRNP*) gene majorly determines the susceptibility of animals to TSEs. However, only limited studies have examined the variation in *PRNP* gene in different Nigerian livestock species. Thus, this study aimed to identify the polymorphism of *PRNP* gene in Nigerian livestock species (including camel, dog, horse, goat, and sheep). We sequenced the open reading frame (ORF) of 65 camels, 31 village dogs and 12 horses from Nigeria and compared with *PRNP* sequences of 886 individuals retrieved from public databases.

**Results:**

All the 994 individuals were assigned into 162 haplotypes. The sheep had the highest number of haplotypes (*n* = 54), and the camel had the lowest (*n* = 7). Phylogenetic tree further confirmed clustering of Nigerian individuals into their various species. We detected five non-synonymous SNPs of *PRNP* comprising of G9A, G10A, C11G, G12C, and T669C shared by all Nigerian livestock species and were in Hardy-Weinberg Equilibrium (HWE). The amino acid changes in these five non-synonymous SNP were all “benign” via Polyphen-2 program. Three SNPs G34C, T699C, and C738G occurred only in Nigerian dogs while C16G, G502A, G503A, and C681A in Nigerian horse. In addition, C50T was detected only in goats and sheep.

**Conclusion:**

Our study serves as the first to simultaneously investigate the polymorphism of *PRNP* gene in Nigerian livestock species and provides relevant information that could be adopted in programs targeted at breeding for prion diseases resistance.

**Supplementary Information:**

The online version contains supplementary material available at 10.1186/s12864-024-10070-2.

## Background

Prion diseases also known as Transmissible spongiform encephalopathies (TSEs) remain one of the deleterious disorders [1], which have affected several animal species [2, 3]. The unique characterization of Prion diseases is the accumulation of an “infectious” abnormal protease-resistant isoform (PrPSc) of cellular prion proteins (PrPC) encrypted by the prion protein (*PRNP*) gene [4]. The prion gene family consist of four members namely prion protein gene (*PRNP*), the prion-like protein gene (*PRND*), the shadow of the prion protein gene (*SPRN*), and the prion-related gene (*PRNT*) [5]. Although only Shadoo (Sho) protein is enclosed by the *SPRN* gene, and its structure is similar to the PrP protein. The *PRND* is nearly situated in 20 kb downstream of the *PRNP* gene, and *PRND* possess a similar structure with PrP [6]. Prion protein genes are highly maintained among mammals [7] and predominantly synthesized in cells of the central nervous system [8]. Although, it is also expressed in different peripheral tissues [9, 10]. Interestingly, in the central nervous system and lymphoid tissues, TSE diseases encompass a neuronal glycoprotein (i.e. Prion protein) PrP^C^ (encoded by *PRNP* gene), which is regenerated into an abnormal protease-resistant protein [11, 12]. Prion diseases are grouped as sporadic, familial, and infectious forms and contains two exons with second one carrying the whole open reading frame (ORF) in humans [13]. It was reported that about 85% of prion diseases in humans are Creutzfeldt–Jakob disease (CJD) while 15% of the prion diseases include familial CJD, Gerstmann–Straussler–Scheinker syndrome (GSS), and fatal familial insomnia (FFI) [14–16]. Another study has reported the nature of the infectious agents- PrP models of resistant species including dog, rabbit and horses to prion diseases [17]. The β2-α2 loop contributes to their protein structural stabilities while salt bridge contributed to structural stability of horse prion protein [18].

Prion diseases are the sole human neurodegenerative disorders with true associates with mammals thereby enabling rodent suitable models to comprehend the mechanisms of disease transmission and pathogenesis. Scrapie is a detrimental neurodegenerative prion malady and has spread across almost all regions worldwide [19, 20] leading to spongiform brain pathology, brain deposition of misfolded among others. Known for over 250 years, scrapie is one of the TSE and encompasses zoonotic bovine spongiform encephalopathy (BSE) in cattle and Creutzfeldt–Jakob disease (CJD) in humans, which are regulated by the prion protein-encoding gene (*PRNP*) [21–23]. It has been reported that the resistance to scrapie is intently regulated by SNPs of the *PRNP* gene and controlled by the prion disease agent [7, 24], and the distribution of SNPs at the ORF of *PRNP* gene in various species was presented [25]. In 1986, classical BSE was first reported in United Kingdom (UK) and has spread through PrP^Sc^ affected meat and bone meal. However, different surveillance approaches have been adopted to prevent utilization of contaminated feed and this has drastically reduced the number of classical BSE cases [26]. It was reported that the insertion of G allele at codon 46 of *SPRN* gene in humans with variant CJD causes a frameshift of this gene and it displays a significant disparity in its distribution between healthy controls and vCJD patients [27]. Moreover, it was reported that somatic mutation of humans’ *PRNP* was predicted to be one of the factors responsible for prion disease [16]. Also, scrapie could affect small ruminants including sheep and goats [28]. In addition, most forms of TSEs affect different mammalian species but display high dominance in ruminants such as scrapie in goats and sheep [29].

Our previous studies revealed that the SNP sites at codons 139 S, 146 S, 154 H, and 193I were presence in Nigerian goats [30] which have been reported to be susceptible to scrapie in goats [28, 31], and also codons 154 H and 171Q susceptible to classical scrapie in sheep [32, 33] detected in Nigerian sheep [34].

*Camelus dromedarius* are vastly found in the semiarid northern part of Nigeria and their estimates is about 289,794 heads [35]. They are basically reared for meat, milk, wool, source of transportation, beauty spectacle, and recreational activities [35]. Nigerian village dogs are one of the major sources of the transmission of infectious diseases [36], horses from Nigeria are useful for entertainment, polo games, ceremonies, research, riding etc. [37]. Nigerian sheep are reared in the drier agro-climatic zones of the country with an estimated population of 27 million [38]. There are four major breeds of Nigerian sheep: Yankasa, Uda, Balami, and West Africa Dwarf [39]. Nigerian goats are hardy, tolerant to trypanosomiasis, and adapt easily to the local ecosystem [40]. There are three main indigenous breeds of Nigerian goats: West African Dwarf (WAD), Sokoto Red and Sahelian [41–43].

Therefore, this study was designed to understand the *PRNP* gene sequence variation in different Nigerian livestock species and provide insight into their resistance to prion diseases. Herein, we combined the *PRNP* sequences of five Nigerian livestock species (camel, dog, horse, sheep, and goat) and analyzed the prion genes. In addition, we retrieved the nucleotide sequences of *PRNPs* from other mammalians for the SNP analyses.

## Results

### Haplotype analysis of the 994 sequences of *PRNP* gene

A total of 994 *PRNP* gene sequences were analyzed, including 108 *de novo* and 886 downloaded from GenBank. All the *PRNP* sequences were assigned into 162 haplotypes (Additional File 1). The sheep had the highest number of haplotypes (*n* = 54), and the camel had the lowest (*n* = 7).

**Fig. 1:**
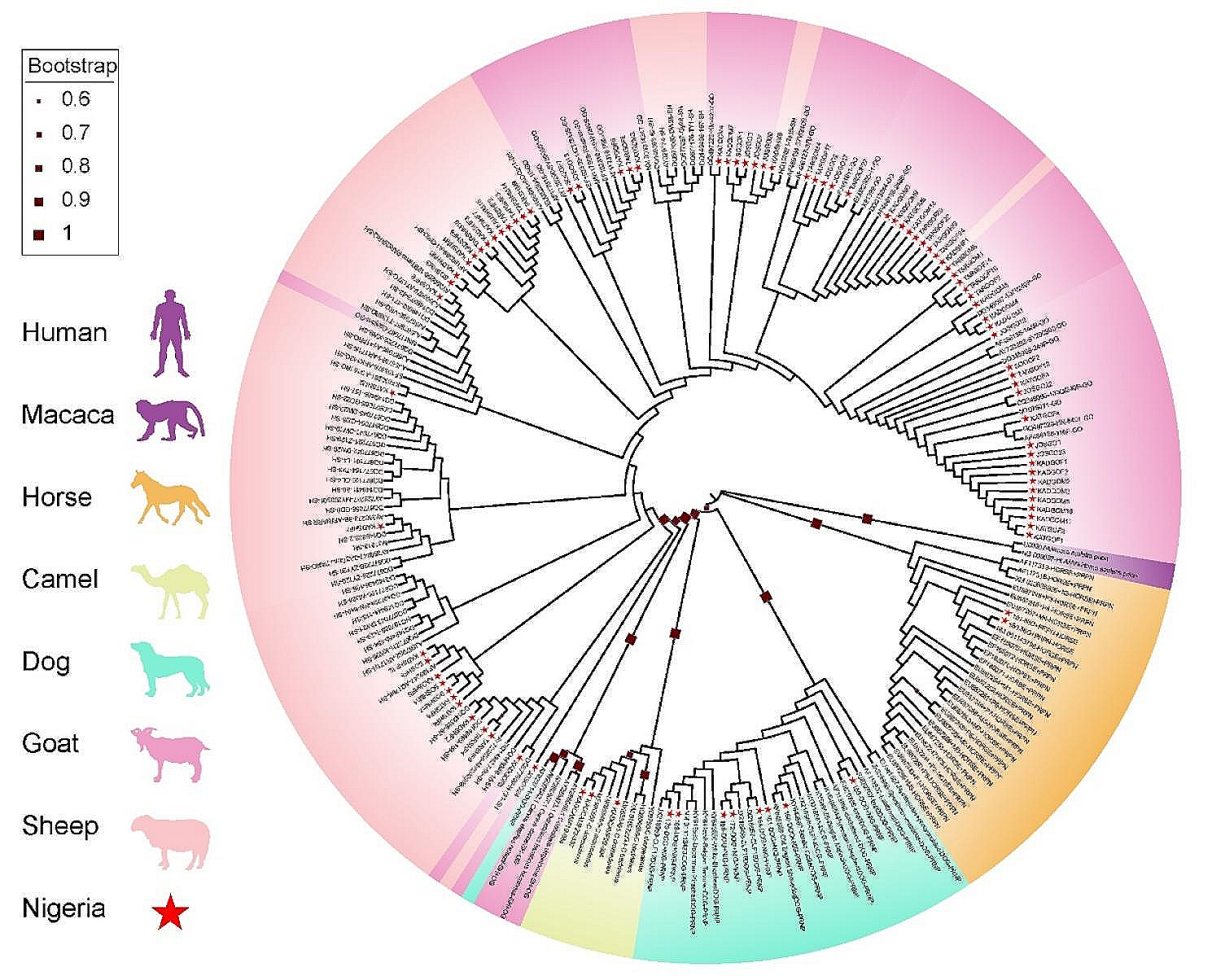
Phylogenetic tree of *PRNP* sequences of Nigerian livestock species and other species

### Phylogenetic tree based on number of haplotypes

Based on the 162 haplotypes, 180 individuals including Nigerian species and those retrieved sequences were selected to construct the phylogenetic tree. Figure 1 showed the phylogenetic tree from the analysis of *PRNP* sequences of five Nigerian species together with reported *PRNP* sequences of *Homo sapiens* and *Macaca mulatta* as outgroups. The phylogenetic tree further confirmed clustering of Nigerian individuals into their various species.

### Single nucleotide polymorphism (SNPs) of *PRNP* gene in the five Nigerian species

We detected five non-synonymous SNPs of *PRNP* namely G9A, G10A, C11G, G12C, and T669C in all Nigerian species considered when combined with nucleotide sequences retrieved from public database (Table 1). Further, we determined the genotype and allele frequencies of the five non-synonymous SNPs detected in Nigerian livestock species and were in Hardy-Weinberg Equilibrium (HWE) (Table 2). Based G34C, T699C, and C738G occurred only in *PRNP* of Nigerian dog while C16G, G502A, G503A, and C681A were identified in Nigerian horse only. In addition, C50T was detected in goat and sheep only. Table 1 shows the non-synonymous SNPs detected in Nigerian livestock species. All SNPs identified in Nigerian livestock species are listed in Additional Table 1.

**Table 1:** The variation of non-synonymous single nucleotide polymorphism (SNPs) of *PRNP* in the five Nigerian livestock species

SNP location	Changes in Amino acid	Species
G9A	M3K	All species
G10A	M3K	All species
C11G	M3K	All species
G12C	A4S	All species
C16G	N5K	Horse
C24T	C8G	goat, sheep
G30T	M10I	All species
G34C	V12L	Dog
C50T	A16V	Goat, sheep
A51G	T17M	Camel, sheep, and goat
C287G	T96S/A	Dog, goat, and sheep
A296G	S98G	21 goat individuals
G340C	M113V	2 sheep individuals
T432C	S144N	Camel
C480G	N160E	3 dog individuals
G501A	M167V	Horse
G502A	M167V	Horse
G503A	M167V	Horse
G505C	D168S	Dog, camel, goat and sheep
A506G	D168S	2 sheep individuals
T508G	D168S	8 sheep individuals
C510T	Y170D	Goat and sheep
C522A	N174K	24 sheep individuals
A524G	N174K	Camel
C534T	H178R	Dog, goat and sheep
T612C T612G	V204M/I	Horse and camel Dog
G618A	M206I	Horse, camel, goat and sheep
G660A	E220Q	1 individual dog
G662A	E220Q	Horse and dog
G663A	R221Q	Horse, camel, goat and sheep
T669C	S223Y	All species
G670G	S223Y	Dog and camel
A678C A678G	Y226F/S	Dog Camel, goat and sheep
T679C	Y226F/S	Horse
C681A	Y227Q	Horse
G693A G693C	S231A	Dog, goat and sheep Camel
T699C	M233A	Dog
G700A	M233A	Dog, camel, goat and sheep
T711C T711G	S237P	Horse, camel, goat and sheep Dog
C738G	F246L	Dog

**Table 2:** Genotype and allele frequencies of the five non-synonymous SNPs detected in Nigerian livestock species

	Genotype frequency, n (%)			Allele frequency, n (%)		HWE
	GG	GA	AA	G	A	
G9A	0 (0.00)	0 (0.00)	366 (1.00)	0 (0.00)	732 (100.00)	> 0.001
M3K						
	GG	GA	AA	G	A	
G10A	0 (0.00)	0 (0.00)	366 (1.00)	0 (0.00)	732 (100.00)	> 0.001
M3K						
	CC	CG	GG	C	G	
C11G	0 (0.00)	0 (0.00)	366 (1.00)	0 (0.00)	732 (100.00)	> 0.001
M3K						
	GG	GC	CC	G	C	
G12C	0 (0.00)	0 (0.00)	366 (1.00)	0 (0.00)	732 (100.00)	> 0.001
A4K						
	TT	TC	CC	T	C	
T669C	0 (0.00)	0 (0.00)	366 (1.00)	0 (0.00)	732 (100.00)	> 0.001
S223Y						

HWE: Hardy Weinberg Equilibrium

### Assessment of the effects of the non-synonymous SNPs

PolyPhen-2 is an online tool used to predict the possible impact of an amino acid replacement caused by nonsynonymous SNPs on the structure and function of proteins [44]. Based on the polymorphism results, effects of the five non-synonymous SNPs common in the five Nigerian livestock species considered were assessed via PolyPhen-2. It was predicted that the amino acid substitution in the five non-synonymous SNPs was benign (Table 3).

**Table 3:** Measurement of the effect of amino-acid substitutions of *PRNP* nonsynonymous SNPs in the five Nigerian livestock species using PolyPhen-2

Position	AA_1_	AA_2_	Score	Prediction
3	M	K	0.000	Benign
4	A	S	0.001	Benign
223	S	Y	0.006	Benign

## Discussion

The polymorphism of the *PRNP* gene plays a great role in the susceptibility of animals to prion protein diseases. In horses, the stability of prion protein is associated with disease progression.

Previous studies have identified single nucleotide mutations at codons 136 (A > V), 154 (R > H), and 171 (R > Q/H) of *PRNP* gene [45, 46]. Interestingly, the variation of amino acids at codons 141 and 154 were reported to be related to various forms of classical scrapie by altering the configuration of prion protein [47, 48]. In addition, the changes in amino acids A to V and Q to R at codons 136 and 171, respectively were reported to increase resistance to scrapie in sheep [20, 45].

The susceptibility of small ruminants (i.e. goat and sheep) to scrapie are affected by the genetic variation of the *PRNP* gene. Goats and sheep share about 99% protein sequence homology for their prion proteins. Although, the fragments of their amino acids associated with scrapie vulnerability are not similar [5, 21]. *PRNP* genes are highly polymorphic in goats [49–52], and the distributions of genotype and haplotype frequencies at codons 139, 146, and 154 were highly associated with vulnerability to scrapie in goats [50, 52].

**Fig. 2:**
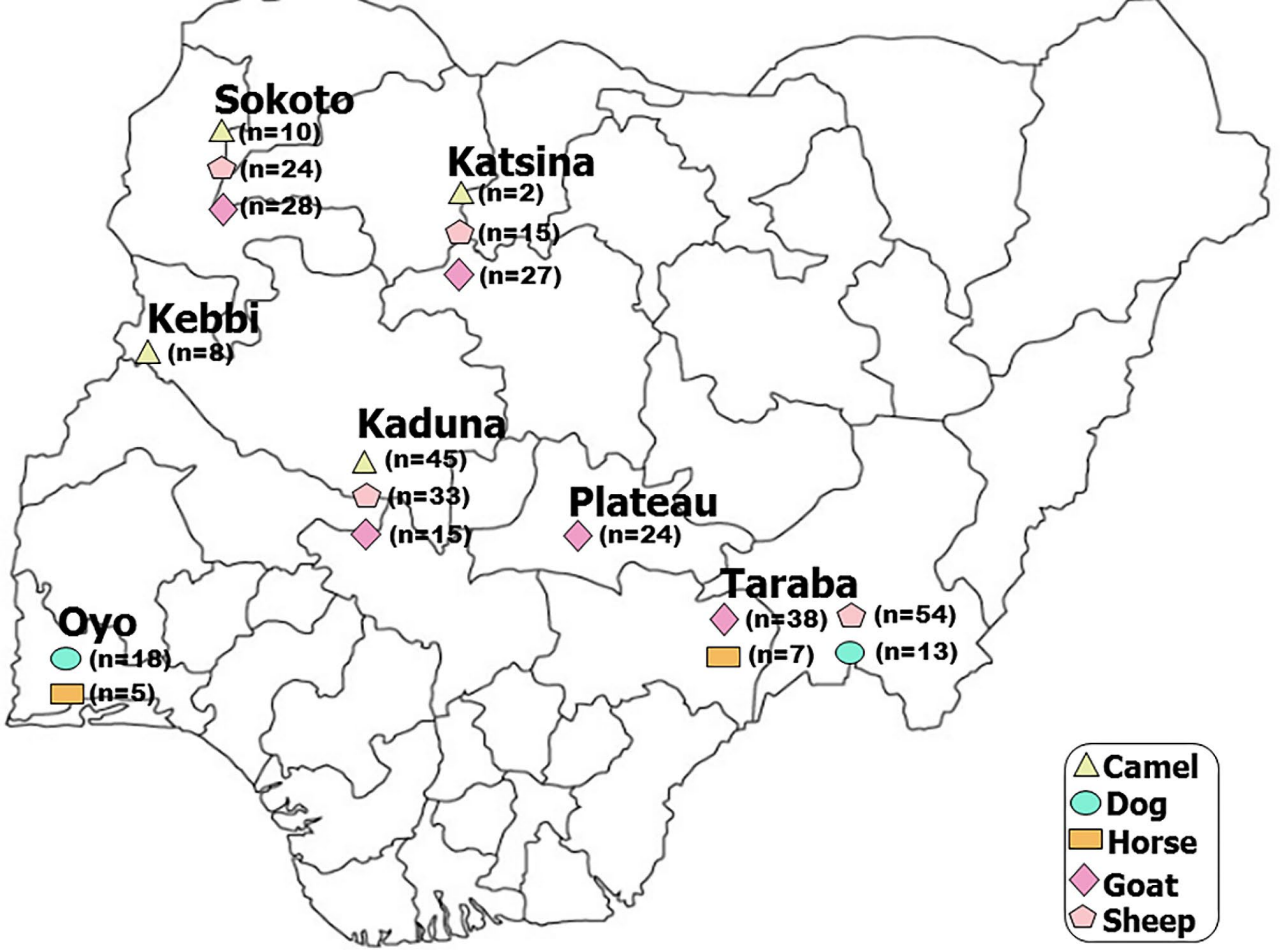
Map of Nigeria showing the sampling locations

We combined the *PRNP* sequences of five Nigerian livestock species (camel, dog, goat, horse, and sheep) and retrieved sequences of human, monkey, camel, dog, goat, sheep, goat, mule deer, Rocky Mountain elk and fallow deer from available databases. We detected 162 haplotypes using the 994 sequences (Additional File 3). Based on the phylogenetic tree when *Homo sapiens* and *Macaca mulatta* are outgroups shows the clustering of Nigerian individuals into their various species (Fig. 2). The sheep had the highest number of haplotypes (*n* = 54), and the camel had the lowest (*n* = 7). We assumed that the Nigerian sheep might be more susceptible to prion related disease than the other four Nigerian livestock species (goat, dog, horse, and camel).

Further, based on the SNP analysis, we detected five non-synonymous SNPs of *PRNP* namely G9A, G10A, C11G, G12C, and T669C in all Nigerian species considered as shown in Table 1. The result shows that the five Nigerian livestock species might be susceptible to prion related diseases. These SNP sites are unique to the Nigerian livestock species considered in this study. Contrarily, previous studies on polymorphism of *PRNP* gene in Nigerian small ruminants, 29 SNPs (14 non-synonymous and 23 novel SNPs) and 19 SNPs (14 non-synonymous SNPs with T718C as a novel SNP) were revealed in Nigerian goats and sheep, respectively [30, 34]. Recent studies have reported low variation in dromedary *PRNP* gene in Egypt and Iran [53, 54]. Two non-synonymous SNPs (G205A and G401A) were identified in *PRNP* gene in Algerian dromedary [55] but not detected in the present study for Nigerian camel. It has been reported that dog are resistance to prion infection due to change of asparagine at codon 163 [56]. In a previous study, the substitutions of amino acids at canine shadow of prion protein (Sho) were all neutral except 70_71DelAA that was deleterious [57]. Previous study on prion protein gene identified only one non-synonymous SNP at c.525 A (N175K) in Thoroughbred horse [58]. Based on PolyPhen-2, it was predicted that the amino acid substitution in the five non-synonymous SNPs common to all the Nigerian livestock species was benign (tolerant).

## Conclusion

This preliminary study aims to examine the single nucleotide polymorphism (SNP) in the open frame region (ORF) of *PRNP* in Nigerian livestock species. Based on our results, we detected five non-synonymous SNPs of *PRNP* namely G9A, G10A, C11G, G12C, and T669C in all Nigerian species. We assumed that Nigerian livestock species might be susceptible to prion related diseases based on these codons identified in our current study. Our preliminary study provides baseline information on prion gene polymorphism in Nigerian livestock species and subsequent studies will examine the functional relationship between clinical signals with prion SNPs from our genomic studies in connection with genotypes of prion protein. In addition, future studies will incorporate large sample size, utilize different coat colors, detect the prevalence of pion protein disease, and functional analyses in *PRNP* gene in Nigerian animals.

## Materials and methods

### Samplings and DNA extraction

We collected about 10 ml of blood samples from 65 camels (25 males and 40 females) from four states in Nigeria including: Kaduna (*n* = 19 males; *n* = 26 females), Sokoto (*n* = 5 males; *n* = 5 females), Kebbi (*n* = 1 male; *n* = 7 females), and Katsina (*n* = 2 females), 31 village dogs from Oyo (*n* = 10 males; *n* = 8 females) and Taraba (*n* = 6 males; *n* = 7 females) and 12 horses from Oyo (*n* = 5) and Taraba (*n* = 7) states (Fig. 2, Additional File 2). During sample collection, we avoided individuals from clustered populations. The whole blood samples were stored at -20 ◦C prior to DNA extraction. Genomic DNA was extracted at Kunming Institute of Zoology, Chinese Academy of Sciences (CAS), using the phenol-chloroform method [59]. We quantified the genomic DNA using the Thermo Scientific™ NanoDrop 2000 spectrophotometer to evaluate its purity. In addition, to check for molecular quality, we ran gel electrophoresis of the genomic DNA using a 2% agarose gel against a 2 Kilobase (kb) DNA ladder marker. In addition, we retrieved nucleotide sequences of *PRNP* of 126 sheep [34] and 132 goats [30] from Nigeria, human, monkey, camel, dog, sheep, goat and horse individuals from database (Additional File 2).

### Polymerase chain reaction (PCR) and DNA sequencing

We amplified the base pairs of the *PRNP* gene in the animals to reveal its variable sites. Primers from each animal were designed based on the nucleotide sequence of the *PRNP* gene retrieved from the NCBI website (Additional Table 2). The 25 µl PCR mixture and sequencing reactions contained 1 µl of genomic DNA,10 pmol of each primer, 2.5mM dNTPs and 5 units of Takara Taq DNA polymerase in a 10 pmol reaction buffer containing 1.5 mM MgCl_2_.

The PCR was carried out in a thermocycler (detailed PCR reactions of each species is presented in Additional File 3. PCR products were purified for sequencing analysis with a QIAquick Gel Extraction Kit (Qiagen, Valencia, California, USA). The PCR products were bidirectionally sequenced using an ABI 3730XL sequencer (Applied Biosystems, Foster City, California, USA).

### Sequences alignment, haplotype, phylogenetic and statistical analyses

The sequences were aligned with MEGA (v.11.0.8) [60]. Nucleotide and amino acid alignments were produced using ClustalW and adjusted manually. We computed genetic distances using MEGA (v.11.0.8). The number of haplotypes in the 994 sequences was analyzed using DnaSP 6 [61]. Further, we determined the distance matrices under the assumptions of Kimura’s two-parameter model and were adopted to infer dendrograms by the neighbor-joining method [62]. The confidence values for individual branches of the resulting tree were determined by bootstrap analysis with 1000 replicates [63]. The allelic and genotypic frequencies of the non-synonymous SNPs common to the five Nigerian species were tested by chi-square test (χ2) or Fisher’s exact test using SPSS version 21.0 (IBM Corp., Armonk, NY).

### Assessment of the effects of the non-synonymous SNPs

The effects of the three (3) nonsynonymous SNPs of *PRNP* gene common to the five Nigerian livestock species were evaluated using PolyPhen- 2 (https://genetics.bwh.harvard.edu/pph2/).

### Electronic supplementary material

Below is the link to the electronic supplementary material.

Supplementary Material 1

Supplementary Material 2

Supplementary Material 3

Supplementary Material 4

Supplementary Material 5

## Data Availability

The nucleotide sequences are available on NCBI with accession numbers: MZ463488 - MZ463499 for horse, MZ463325 - MZ463355 for dog, and OK041226 - OK041290 for camel.

## Abbreviations

*PRNP*: Prion protein
TSEs: Transmissible spongiform encephalopathies
PrPSc: Protease-resistant isoform
PrPC: Cellular prion proteins
*SPRN*: Shadow of the prion protein gene
*PRNT*: The prion-related gene
CJD: Creutzfeldt–Jakob disease
SNPs: Single Nucleotide Polymorphism
WAD: West African Dwarf
ORF: Open reading frame
