# Supplementary material for: Single nucleotide polymorphisms (SNPs) in the open reading frame (ORF) of prion protein gene (PRNP) in Nigerian livestock species
Source: BMC Genomics. 2024 Feb 14;25:177. doi: 10.1186/s12864-024-10070-2 (PMC10865551; doi:10.1186/s12864-024-10070-2)
Supplement: Supplementary file 2 — Supplementary Material 2 [file 12864_2024_10070_MOESM2_ESM.docx]

Additional File 3: PCR reactions for each animal

**Camel**

5 minutes at 96°C; 30 seconds at 96°C, 15 seconds at 57°C, 1 minute 30 seconds at 72°C for 40 cycles, and a final extension of 4 minutes at 72°C.

**Dog**

95 °C for 2 mins, 34 cycles of denaturation at 95 °C for 20 s, 62 °C for 30 s, 72 °C for 1 min 30 sec, and a final extension of 72 °C at 5 mins.

**Horse**

Initial denaturation at 94 °C for 2 mins, 35 cycles of denaturation at 94 °C for 45s, 59 °C for 45s, 72 °C for 90s, and a final extension of 72 °C at 5 mins.
