# Supplementary material for: Single nucleotide polymorphisms (SNPs) in the open reading frame (ORF) of prion protein gene (PRNP) in Nigerian livestock species
Source: BMC Genomics. 2024 Feb 14;25:177. doi: 10.1186/s12864-024-10070-2 (PMC10865551; doi:10.1186/s12864-024-10070-2)
Supplement: Supplementary file 3 — Supplementary Material 3 [file 12864_2024_10070_MOESM3_ESM.docx]

Additional Table 1: The single nucleotide polymorphism (SNPs) of *PRNP* in five Nigerian livestock species

| **SNP location** | **Changes in Amino acid** | **Species** |
| --- | --- | --- |
| GA1 |  | All species |
| C3G | V1M | All species |
| A4G | V1M | All species |
| TG6 | I2V | All species |
| G8A | I2V | All species |
| G9A | M3K | All species |
| G10A | M3K | All species |
| C11G | M3K | All species |
| G12C | A4S | All species |
| C16G | N5K | Horse |
| T18A | L6V | All species |
| T22G | 7G | All species |
| C24T | C8G | Sheep, goat |
| G30T | M10I | All species |
| G34C | V12L | Dog |
| C47T | 15V | Camel |
| C50T | A16V | Goat, sheep |
| A51G | T17M | Camel, sheep and goat |
| C61G | 20D | All species |
| C66G | 22G | Horse |
| C69G | 23L | Horse |
| C81A | 27R | Goat and sheep |
| G84A | 28P | Camel, goat and sheep |
| G87A | 29K | Sheep and goat |
| T105A | 35T | Dog |
| C111G | 37G | All species |
| G123A | 41P | Dog, camel and 11 individuals from goat |
| G126C  G126A | 42G | Dog  Goat and sheep |
| C132T | 44G | Dog |
| C136T | 46S | Camel, goat and sheep |
| C153T | 51Y | Camel, goat and sheep |
| T159C | 53P | Horse, dog and camel |
| C165A | 55G | Camel, goat and sheep |
| T168G | 56G | Camel |
| T171C | 57G | Camel |
| G180T | 60G | All species |
| G183A | 61Q | Horse |
| T186C | 62P | All species |
| T189C | 63H | Camel |
| T192A | 64G | Camel, sheep and goat |
| T195A | 65G | Camel |
| G204T  G204C | 68G | Horse, dog and camel  Sheep and goat |
| G207A | 69Q | Camel, goat and sheep |
| T210C | 70P | Camel |
| T213C | 71H | Camel |
| T216A | 72G | All species |
| T219A | 73G | Dog and camel |
| G228T | 76G | All species |
| C234G | 78P | Horse |
| T237C | 79H | Dog and camel |
| T240A | 80G | Camel |
| T243C | 81G | Camel |
| A252C | 82G | Camel |
| T258C  T258A | 86P | Horse and camel  Dog, goat and sheep |
| T267A | 89G | Horse, camel, goat and sheep |
| C270T | 90G | Dog |
| C285T | 95G | Dog, camel, goat and sheep |
| A286G  A286T | 95G | Horse  Camel |
| C287G | T96S/A | Dog, goat and sheep |
| G291C | 97H | Horse |
| A292G | 97H | Horse, camel and 10 goat individuals |
| A296G | S98G | 21 goat individuals |
| A301G | 100W | Dog |
| A302G | 100W | Dog |
| G309C | 103P | All species |
| G311A | 103P | Dog |
| A318C  A318G | 106P | Dog  Camel |
| A326G | 108T | Camel |
| C336T | 112H | Horse, goat and sheep |
| A337G | 112H | All species |
| G340C | M113V | 2 sheep individuals |
| T342A | 114A | All species |
| T345A | 115G | All species |
| T348C | 116A | Dog |
| A351G | 117A | Horse, camel, goat and sheep |
| T357C | 119A | Dog |
| G360A | 120G | Goat and sheep |
| A363G | 121A | Dog |
| G366A | 122V | Dog |
| G369T  G369A | 123V | Horse  Dog and camel |
| T378C | 126L | Horse and camel |
| C381T | 127G | Dog, goat and sheep |
| A396T | 132G | Horse, dog and camel |
| C408C | 136S | 118 goat individuals |
| C414A  C414T | 138P | Dog  Goat and sheep |
| A415C | 138P | All species |
| C417T | 139T | Camel, goat and sheep |
| A420T | 140I | Camel and dog |
| A422G | 140I | 16 sheep individuals |
| C426T | 142F | All species |
| G431A | 143G | All species |
| T432C | S144N | Camel |
| C445G | 148D | 1 sheep individual |
| G452A | 150Y | 40 goat and 5 sheep individuals |
| T456A | 152R | Camel |
| A459G | 153E | Dog |
| C466T | 155M | All species |
| T471C | 157R | Dog |
| A478G | 159P | Dog |
| C480G | N160E | 3 dog individuals |
| G486A | 162V | Dog |
| A493C | 164Y | Dog |
| C494A | 164Y | Camel |
| G495A | 165R | Goat and sheep |
| C498G  C498A | 166P | Horse  Dog, camel, goat and sheep |
| A499G | 166P | All species |
| G501A | M167V | Horse |
| G502A | M167V | Horse |
| G503A | M167V | Horse |
| G505C | D168S | Dog, camel, goat and sheep |
| A506G | D168S | 2 sheep individuals |
| T508G | D168S | Eight sheep individuals |
| C510T | Y170D | Goat and sheep |
| C513T | 171S | Goat and sheep |
| C522A | N174K | 24 sheep individuals |
| A524G | N174K | Camel |
| T528C | 176F | Camel |
| A533G | 177V | Dog |
| C534T | H178R | Dog, goat and sheep |
| C540T | 180C | Goat and sheep |
| T546C | 181V | All species |
| A552G  A552T | 184N | Horse  Dog |
| A553G | 184N | All species |
| G558A | 186K | Camel |
| C559G | 186K | 1 goat individuals |
| G561A | 187Q | Goat and sheep |
| G567A | 189T | 4 individual horse, dog, goat and sheep |
| C570G | 190V | Dog |
| C572T | 190V | Goat |
| A576C | 192T | All species |
| C600A | 200T | Dog |
| G603A | 201E | Goat and sheep |
| C606T | 202T | Goat and sheep |
| G610A | 203D | Dog, goat and sheep |
| T612C  T612G | V204M/I | Horse and camel  Dog |
| G618A | M206I | Horse, camel, goat and sheep |
| G626A | 208E | 1 individual goat |
| C627T  C627A | 209R | Dog  Goat and sheep |
| G630A | 210V | Camel |
| G631A | 210V | 3 goat individual |
| T633G | 211V | Horse |
| G639A | 213Q | Camel, goat and sheep |
| T645C | 215C | Horse, camel, goat and sheep |
| A646G | 215C | Dog |
| C648T | 216I | 1 individual goat |
| G658C | 219Y | All species |
| G660A | E220Q | 1 individual dog |
| G662A | E220Q | Horse and dog |
| G663A | R221Q | Horse, camel, goat and sheep |
| A666G | 222E | Horse |
| C668A | 222E | Horse and camel |
| T669C | S223Y | All species |
| G670G | S223Y | Dog and camel |
| C675T | 225A | All species |
| A677C | 225A | Camel |
| A678C  A678G | Y226F/S | Dog  Camel, goat and sheep |
| T679C | Y226F/S | Horse |
| C681A | Y227Q | Horse |
| C682G | 227Y | Camel |
| A683G | 227Y | Camel |
| G684A  G864C | 228Q | Horse, goat and sheep  Camel |
| A685C | 228Q | 2 goat and 37 sheep individuals |
| A687G | 229R | Goat and sheep |
| A690G | 230Q | All species |
| A691G | 230Q | All species |
| G693A  G693C | S231A | Dog, goat and sheep  Camel |
| A697G | 232S | All species |
| T698C | 232S | Dog |
| T699C | M233A | Dog |
| G700A | M233A | Dog, camel, goat and sheep |
| C705T | 235F | Goat and sheep |
| C708T | 236S | Goat and sheep |
| T709C | 236S | Dog and 105 goat individuals |
| T711C | S237P | Horse, camel, goat and sheep  Dog |
| A714G  A714T | 238P | Horse and dog  Camel, goat and sheep |
| A721G | 240V | Horse |
| G729C | 243L | All species |
| T735G | 245S | Dog |
| T736C | 245S | Dog |
| C738G | F246L | Dog |
| C744T | 248I | All species |
| T745C | 248I | Dog |
| C747T | 249F | Goat and sheep |
| C748T | 249F | Dog |
| G750C | 250L | Camel, goat and sheep |
| G756A | 252V | Goat and sheep |
