# Supplementary material for: Single nucleotide polymorphisms (SNPs) in the open reading frame (ORF) of prion protein gene (PRNP) in Nigerian livestock species
Source: BMC Genomics. 2024 Feb 14;25:177. doi: 10.1186/s12864-024-10070-2 (PMC10865551; doi:10.1186/s12864-024-10070-2)
Supplement: Supplementary file 4 — Supplementary Material 4 [file 12864_2024_10070_MOESM4_ESM.docx]

Additional Table 2: The details of the primers

| Species | Primers |  | NCBI Gene ID | Base pairs (bp) | | | References |
| --- | --- | --- | --- | --- | --- | --- | --- |
| Camel | Forward | GCTGACACCCTCTTTATTTTGCAG | Y09760 | | 764 |  | |
|  | Reverse | GATTAAGAAGATAATGAAAACAGGAAG |  | |  |  | |
| Dog | Forward | TGTGCAGATGTTCTCGCTGT | 485783 | | 774 | (Kim et al., 2021) | |
|  | Reverse | GAAGCGGGAATGAGACACCA |  | |  |  | |
| Horse | Forward | AGAAGTGCAGAGTGTGACATGC | NM_001143798 | | 836 | (Kim et al., 2020) | |
|  | Reverse | CAAGCGTATTAGCCTACGGGTG |  | |  |  | |
|  | Forward | GCCCGTTGCAGCTTCTTATCT |  | |  |  | |
|  | Reverse | GCTGGAGGAGAGAAGTGGGAT |  | |  |  | |
