# Supplementary material for: Single nucleotide polymorphisms (SNPs) in the open reading frame (ORF) of prion protein gene (PRNP) in Nigerian livestock species
Source: BMC Genomics. 2024 Feb 14;25:177. doi: 10.1186/s12864-024-10070-2 (PMC10865551; doi:10.1186/s12864-024-10070-2)
Supplement: Supplementary file 5 — Supplementary Material 5 [file 12864_2024_10070_MOESM5_ESM.docx]

Additional File 1: The 162 haplotypes detected in the 994 sequences of prion protein (*PRNP*) gene in different species

| S/No | Size | HT name | *Homo sapiens* | *Macaca mulatta* | Horse | Dog | camel | Goat | Sheep | *Odocoileus hemionus hemionus* | *Cervus elaphus nelsoni* | *Odocoileus virginianus* | *Cervus dama* |
| --- | --- | --- | --- | --- | --- | --- | --- | --- | --- | --- | --- | --- | --- |
| 1 | 1 | NG_009087-HUMAN-Homo | 1 | 0 | 0 | 0 | 0 | 0 | 0 | 0 | 0 | 0 | 0 |
| 2 | 1 | U08307-Macaca_mulatt | 0 | 1 | 0 | 0 | 0 | 0 | 0 | 0 | 0 | 0 | 0 |
| 3 | 12 | NM_001143798-HORSE+P | 0 | 0 | 12 | 0 | 0 | 0 | 0 | 0 | 0 | 0 | 0 |
| 4 | 1 | EU887260+M7-HORSE+PR | 0 | 0 | 1 | 0 | 0 | 0 | 0 | 0 | 0 | 0 | 0 |
| 5 | 1 | EU887259+M6-HORSE+PR | 0 | 0 | 1 | 0 | 0 | 0 | 0 | 0 | 0 | 0 | 0 |
| 6 | 1 | EU887257+M4-HORSE+PR | 0 | 0 | 1 | 0 | 0 | 0 | 0 | 0 | 0 | 0 | 0 |
| 7 | 2 | EU887257+M4-HORSE+PR | 0 | 0 | 2 | 0 | 0 | 0 | 0 | 0 | 0 | 0 | 0 |
| 8 | 1 | EU887256+M3-HORSE+PR | 0 | 0 | 1 | 0 | 0 | 0 | 0 | 0 | 0 | 0 | 0 |
| 9 | 1 | EU887255+M2-HORSE+PR | 0 | 0 | 1 | 0 | 0 | 0 | 0 | 0 | 0 | 0 | 0 |
| 10 | 1 | EU887254+M1-HORSE+PR | 0 | 0 | 1 | 0 | 0 | 0 | 0 | 0 | 0 | 0 | 0 |
| 11 | 3 | XM_023625926+X3-HOR | 0 | 0 | 3 | 0 | 0 | 0 | 0 | 0 | 0 | 0 | 0 |
| 12 | 2 | AF117318-HORSE+PRPN | 0 | 0 | 2 | 0 | 0 | 0 | 0 | 0 | 0 | 0 | 0 |
| 13 | 1 | EF165072-HORSE+PRPN | 0 | 0 | 1 | 0 | 0 | 0 | 0 | 0 | 0 | 0 | 0 |
| 14 | 1 | EF165071-HORSE+PRPN] | 0 | 0 | 1 | 0 | 0 | 0 | 0 | 0 | 0 | 0 | 0 |
| 15 | 1 | EF165070-HORSE+PRPN | 0 | 0 | 1 | 0 | 0 | 0 | 0 | 0 | 0 | 0 | 0 |
| 16 | 1 | EU887253-HORSE+PRPN | 0 | 0 | 1 | 0 | 0 | 0 | 0 | 0 | 0 | 0 | 0 |
| 17 | 1 | EU887252-HORSE+PRPN | 0 | 0 | 1 | 0 | 0 | 0 | 0 | 0 | 0 | 0 | 0 |
| 18 | 1 | EU887251+P9-HORSE+PR | 0 | 0 | 1 | 0 | 0 | 0 | 0 | 0 | 0 | 0 | 0 |
| 19 | 4 | 191-NIG+PRPN | 0 | 0 | 4 | 0 | 0 | 0 | 0 | 0 | 0 | 0 | 0 |
| 20 | 1 | EU887250+P8-HORSE+PR | 0 | 0 | 1 | 0 | 0 | 0 | 0 | 0 | 0 | 0 | 0 |
| 21 | 1 | EU887249+P7-HORSE+PR | 0 | 0 | 1 | 0 | 0 | 0 | 0 | 0 | 0 | 0 | 0 |
| 22 | 1 | EU887248+P6-HORSE+PR | 0 | 0 | 1 | 0 | 0 | 0 | 0 | 0 | 0 | 0 | 0 |
| 23 | 1 | EU887246+P4-HORSE+PR | 0 | 0 | 1 | 0 | 0 | 0 | 0 | 0 | 0 | 0 | 0 |
| 24 | 1 | EU887247+P5-HORSE+PR | 0 | 0 | 1 | 0 | 0 | 0 | 0 | 0 | 0 | 0 | 0 |
| 25 | 1 | EU887245+P3-HORSE+PR | 0 | 0 | 1 | 0 | 0 | 0 | 0 | 0 | 0 | 0 | 0 |
| 26 | 1 | EU887244+P2-HORSE+PR | 0 | 0 | 1 | 0 | 0 | 0 | 0 | 0 | 0 | 0 | 0 |
| 27 | 1 | EU887243+P1-HORSE+PR | 0 | 0 | 1 | 0 | 0 | 0 | 0 | 0 | 0 | 0 | 0 |
| 28 | 1 | AF117312-HORSE+PRPN | 0 | 0 | 1 | 0 | 0 | 0 | 0 | 0 | 0 | 0 | 0 |
| 29 | 1 | EF165075-HORSE+PRPN | 0 | 0 | 1 | 0 | 0 | 0 | 0 | 0 | 0 | 0 | 0 |
| 30 | 2 | NM_001013423-DOG-PRN | 0 | 0 | 0 | 2 | 0 | 0 | 0 | 0 | 0 | 0 | 0 |
| 31 | 4 | JX218959-CLF17DOG-PR | 0 | 0 | 0 | 4 | 0 | 0 | 0 | 0 | 0 | 0 | 0 |
| 32 | 4 | JX218958-CLF16DOG-PR | 0 | 0 | 0 | 4 | 0 | 0 | 0 | 0 | 0 | 0 | 0 |
| 33 | 8 | JX218957-CLF15DOG-PR | 0 | 0 | 0 | 8 | 0 | 0 | 0 | 0 | 0 | 0 | 0 |
| 34 | 1 | JX218956-CLF14DOG-PR] | 0 | 0 | 0 | 1 | 0 | 0 | 0 | 0 | 0 | 0 | 0 |
| 35 | 1 | FJ870767-tmp2DOG-PRN | 0 | 0 | 0 | 1 | 0 | 0 | 0 | 0 | 0 | 0 | 0 |
| 36 | 1 | FJ870766-tmp1DOG-PRN | 0 | 0 | 0 | 1 | 0 | 0 | 0 | 0 | 0 | 0 | 0 |
| 37 | 2 | KY649563-Doberman_Pi | 0 | 0 | 0 | 2 | 0 | 0 | 0 | 0 | 0 | 0 | 0 |
| 38 | 26 | KY649560-Border_Coll KY649559-Old_English | 0 | 0 | 0 | 26 | 0 | 0 | 0 | 0 | 0 | 0 | 0 |
| 39 | 1 | KY649558-Belgian_Ter | 0 | 0 | 0 | 1 | 0 | 0 | 0 | 0 | 0 | 0 | 0 |
| 40 | 1 | KY649556-White_Sheph | 0 | 0 | 0 | 1 | 0 | 0 | 0 | 0 | 0 | 0 | 0 |
| 41 | 2 | KY649555-Belgian_Mal | 0 | 0 | 0 | 2 | 0 | 0 | 0 | 0 | 0 | 0 | 0 |
| 42 | 1 | KY649554-Pyrenean_Sh | 0 | 0 | 0 | 1 | 0 | 0 | 0 | 0 | 0 | 0 | 0 |
| 43 | 1 | MG018983-DOG-PRNP | 0 | 0 | 0 | 1 | 0 | 0 | 0 | 0 | 0 | 0 | 0 |
| 44 | 1 | AF113938-dachshund-D | 0 | 0 | 0 | 1 | 0 | 0 | 0 | 0 | 0 | 0 | 0 |
| 45 | 1 | AF113937-hallstromii | 0 | 0 | 0 | 1 | 0 | 0 | 0 | 0 | 0 | 0 | 0 |
| 46 | 1 | AF022714-DOG-PRNP | 0 | 0 | 0 | 1 | 0 | 0 | 0 | 0 | 0 | 0 | 0 |
| 47 | 1 | JX218950-Speothos+ve | 0 | 0 | 0 | 1 | 0 | 0 | 0 | 0 | 0 | 0 | 0 |
| 48 | 1 | [EU341507-Nyctereutes | 0 | 0 | 0 | 1 | 0 | 0 | 0 | 0 | 0 | 0 | 0 |
| 49 | 1 | 158-DOG+NIG-PRNP | 0 | 0 | 0 | 1 | 0 | 0 | 0 | 0 | 0 | 0 | 0 |
| 50 | 1 | 159-DOG+NIG-PRNP | 0 | 0 | 0 | 1 | 0 | 0 | 0 | 0 | 0 | 0 | 0 |
| 51 | 2 | 161-DOG+NIG-PRNP | 0 | 0 | 0 | 2 | 0 | 0 | 0 | 0 | 0 | 0 | 0 |
| 52 | 1 | 169-DOG+NIG-PRNP | 0 | 0 | 0 | 1 | 0 | 0 | 0 | 0 | 0 | 0 | 0 |
| 53 | 17 | MF990557-C_dromedari | 0 | 0 | 0 | 0 | 17 | 0 | 0 | 0 | 0 | 0 | 0 |
| 54 | 16 | HQ204566-C_bactrianu | 0 | 0 | 0 | 0 | 16 | 0 | 0 | 0 | 0 | 0 | 0 |
| 55 | 71 | MF990557-C_dromedari | 0 | 0 | 0 | 0 | 71 | 0 | 0 | 0 | 0 | 0 | 0 |
| 56 | 1 | KADCAMF20-394 | 0 | 0 | 0 | 0 | 1 | 0 | 0 | 0 | 0 | 0 | 0 |
| 57 | 1 | MK655460-C_dromedari | 0 | 0 | 0 | 0 | 1 | 0 | 0 | 0 | 0 | 0 | 0 |
| 58 | 1 | MK655461-C_dromedari | 0 | 0 | 0 | 0 | 1 | 0 | 0 | 0 | 0 | 0 | 0 |
| 59 | 1 | Y09760-C_dromedarius | 0 | 0 | 0 | 0 | 1 | 0 | 0 | 0 | 0 | 0 | 0 |
| 60 | 53 | MT076071-GO | 0 | 0 | 0 | 0 | 0 | 53 | 0 | 0 | 0 | 0 | 0 |
| 61 | 2 | NM_001314247-GO | 0 | 0 | 0 | 0 | 0 | 2 | 0 | 0 | 0 | 0 | 0 |
| 62 | 1 | DQ013244-GO | 0 | 0 | 0 | 0 | 0 | 1 | 0 | 0 | 0 | 0 | 0 |
| 63 | 2 | AY723292-SY200303-GO | 0 | 0 | 0 | 0 | 0 | 2 | 0 | 0 | 0 | 0 | 0 |
| 64 | 1 | AY723290-SY200301-GO | 0 | 0 | 0 | 0 | 0 | 1 | 0 | 0 | 0 | 0 | 0 |
| 65 | 1 | DQ345068-240P-GO | 0 | 0 | 0 | 0 | 0 | 1 | 0 | 0 | 0 | 0 | 0 |
| 66 | 3 | DQ345067-137I/240P-G | 0 | 0 | 0 | 0 | 0 | 3 | 0 | 0 | 0 | 0 | 0 |
| 67 | 1 | DQ345066-133Q/240P-G | 0 | 0 | 0 | 0 | 0 | 1 | 0 | 0 | 0 | 0 | 0 |
| 68 | 1 | EF192309-142T/240S-G | 0 | 0 | 0 | 0 | 0 | 1 | 0 | 0 | 0 | 0 | 0 |
| 69 | 1 | EF192310.-194P/240S- | 0 | 0 | 0 | 0 | 0 | 1 | 0 | 0 | 0 | 0 | 0 |
| 70 | 1 | MN117968-Raighar-GO | 0 | 0 | 0 | 0 | 0 | 1 | 0 | 0 | 0 | 0 | 0 |
| 71 | 193 | MN117967-Ganjam-GO | 0 | 0 | 0 | 0 | 0 | 193 | 0 | 0 | 0 | 0 | 0 |
| 72 | 1 | AF486138-110P-GO | 0 | 0 | 0 | 0 | 0 | 1 | 0 | 0 | 0 | 0 | 0 |
| 73 | 1 | AF486137-37V-GO | 0 | 0 | 0 | 0 | 0 | 1 | 0 | 0 | 0 | 0 | 0 |
| 74 | 10 | AF486136-240S-GO | 0 | 0 | 0 | 0 | 0 | 10 | 0 | 0 | 0 | 0 | 0 |
| 75 | 1 | AF486135-143R-GO | 0 | 0 | 0 | 0 | 0 | 1 | 0 | 0 | 0 | 0 | 0 |
| 76 | 1 | AF486134-37V/240S-GO | 0 | 0 | 0 | 0 | 0 | 1 | 0 | 0 | 0 | 0 | 0 |
| 77 | 4 | AF117316-PBL-GO | 0 | 0 | 0 | 0 | 0 | 4 | 0 | 0 | 0 | 0 | 0 |
| 78 | 1 | AF117315-GO | 0 | 0 | 0 | 0 | 0 | 1 | 0 | 0 | 0 | 0 | 0 |
| 79 | 20 | JOSGO2 | 0 | 0 | 0 | 0 | 0 | 20 | 0 | 0 | 0 | 0 | 0 |
| 80 | 23 | JOSGO3 | 0 | 0 | 0 | 0 | 0 | 23 | 0 | 0 | 0 | 0 | 0 |
| 81 | 6 | JOSGO7 | 0 | 0 | 0 | 0 | 0 | 6 | 0 | 0 | 0 | 0 | 0 |
| 82 | 1 | JOSGO12 | 0 | 0 | 0 | 0 | 0 | 1 | 0 | 0 | 0 | 0 | 0 |
| 83 | 1 | JOSGO13 | 0 | 0 | 0 | 0 | 0 | 1 | 0 | 0 | 0 | 0 | 0 |
| 84 | 3 | JOSGO20 | 0 | 0 | 0 | 0 | 0 | 3 | 0 | 0 | 0 | 0 | 0 |
| 85 | 1 | JOSGO22 | 0 | 0 | 0 | 0 | 0 | 1 | 0 | 0 | 0 | 0 | 0 |
| 86 | 1 | JOSGO24 | 0 | 0 | 0 | 0 | 0 | 1 | 0 | 0 | 0 | 0 | 0 |
| 87 | 3 | KADGOF3 | 0 | 0 | 0 | 0 | 0 | 3 | 0 | 0 | 0 | 0 | 0 |
| 88 | 2 | KADGOM7 | 0 | 0 | 0 | 0 | 0 | 2 | 0 | 0 | 0 | 0 | 0 |
| 89 | 1 | KADGOM8 | 0 | 0 | 0 | 0 | 0 | 1 | 0 | 0 | 0 | 0 | 0 |
| 90 | 3 | KATGOM4 | 0 | 0 | 0 | 0 | 0 | 3 | 0 | 0 | 0 | 0 | 0 |
| 91 | 1 | SOGOF2 | 0 | 0 | 0 | 0 | 0 | 1 | 0 | 0 | 0 | 0 | 0 |
| 92 | 1 | SOGOF7 | 0 | 0 | 0 | 0 | 0 | 1 | 0 | 0 | 0 | 0 | 0 |
| 93 | 1 | TARGOF2 | 0 | 0 | 0 | 0 | 0 | 1 | 0 | 0 | 0 | 0 | 0 |
| 94 | 1 | TARGOF10 | 0 | 0 | 0 | 0 | 0 | 1 | 0 | 0 | 0 | 0 | 0 |
| 95 | 1 | TARGOF12 | 0 | 0 | 0 | 0 | 0 | 1 | 0 | 0 | 0 | 0 | 0 |
| 96 | 2 | TARGOF14 | 0 | 0 | 0 | 0 | 0 | 2 | 0 | 0 | 0 | 0 | 0 |
| 97 | 1 | TARGOF17 | 0 | 0 | 0 | 0 | 0 | 1 | 0 | 0 | 0 | 0 | 0 |
| 98 | 1 | TARGOM1 | 0 | 0 | 0 | 0 | 0 | 1 | 0 | 0 | 0 | 0 | 0 |
| 99 | 1 | TARGOM4 | 0 | 0 | 0 | 0 | 0 | 1 | 0 | 0 | 0 | 0 | 0 |
| 100 | 1 | X91999-GO | 0 | 0 | 0 | 0 | 0 | 1 | 0 | 0 | 0 | 0 | 0 |
| 101 | 1 | EU032305-11-GO | 0 | 0 | 0 | 0 | 0 | 1 | 0 | 0 | 0 | 0 | 0 |
| 102 | 1 | EU032304-10-GO | 0 | 0 | 0 | 0 | 0 | 1 | 0 | 0 | 0 | 0 | 0 |
| 103 | 1 | GQ497228-KM-9207-GO | 0 | 0 | 0 | 0 | 0 | 1 | 0 | 0 | 0 | 0 | 0 |
| 104 | 2 | GQ497223-HN-6401-GO | 0 | 0 | 0 | 0 | 0 | 2 | 0 | 0 | 0 | 0 | 0 |
| 105 | 50 | M31313-SH | 0 | 0 | 0 | 0 | 0 | 0 | 50 | 0 | 0 | 0 | 0 |
| 106 | 29 | HQ197671-Ts15-SH | 0 | 0 | 0 | 0 | 0 | 0 | 29 | 0 | 0 | 0 | 0 |
| 107 | 31 | HQ197669-Ts20-SH | 0 | 0 | 0 | 0 | 0 | 0 | 31 | 0 | 0 | 0 | 0 |
| 108 | 16 | AF195247-AQ176K-SH | 0 | 0 | 0 | 0 | 0 | 0 | 16 | 0 | 0 | 0 | 0 |
| 109 | 6 | AJ567986-A141FRQ-SH | 0 | 0 | 0 | 0 | 0 | 0 | 6 | 0 | 0 | 0 | 0 |
| 110 | 3 | AJ567988-VRQ-SH | 0 | 0 | 0 | 0 | 0 | 0 | 3 | 0 | 0 | 0 | 0 |
| 111 | 1 | AJ567987-T136RQ-SH | 0 | 0 | 0 | 0 | 0 | 0 | 1 | 0 | 0 | 0 | 0 |
| 112 | 1 | AJ567982-AR171H-SH | 0 | 0 | 0 | 0 | 0 | 0 | 1 | 0 | 0 | 0 | 0 |
| 113 | 7 | AJ567981-AR171K-SH | 0 | 0 | 0 | 0 | 0 | 0 | 7 | 0 | 0 | 0 | 0 |
| 114 | 42 | DQ149499-168-SH | 0 | 0 | 0 | 0 | 0 | 0 | 42 | 0 | 0 | 0 | 0 |
| 115 | 43 | DQ149498-167-SH | 0 | 0 | 0 | 0 | 0 | 0 | 43 | 0 | 0 | 0 | 0 |
| 116 | 24 | DQ149496-165-SH | 0 | 0 | 0 | 0 | 0 | 0 | 24 | 0 | 0 | 0 | 0 |
| 117 | 64 | DQ149488-157-SH | 0 | 0 | 0 | 0 | 0 | 0 | 64 | 0 | 0 | 0 | 0 |
| 118 | 4 | DQ149487-156-SH | 0 | 0 | 0 | 0 | 0 | 0 | 4 | 0 | 0 | 0 | 0 |
| 119 | 1 | DQ149444-113-SH | 0 | 0 | 0 | 0 | 0 | 0 | 1 | 0 | 0 | 0 | 0 |
| 120 | 1 | DQ149427-96-SH | 0 | 0 | 0 | 0 | 0 | 0 | 1 | 0 | 0 | 0 | 0 |
| 121 | 8 | DQ149393-62-SH | 0 | 0 | 0 | 0 | 0 | 0 | 8 | 0 | 0 | 0 | 0 |
| 122 | 6 | DQ149393-62-SH | 0 | 0 | 0 | 0 | 0 | 0 | 6 | 0 | 0 | 0 | 0 |
| 123 | 4 | DQ149346-15-SH | 0 | 0 | 0 | 0 | 0 | 0 | 4 | 0 | 0 | 0 | 0 |
| 124 | 1 | DQ149333-2-SH | 0 | 0 | 0 | 0 | 0 | 0 | 1 | 0 | 0 | 0 | 0 |
| 125 | 1 | AY723288-MY200302-SH | 0 | 0 | 0 | 0 | 0 | 0 | 1 | 0 | 0 | 0 | 0 |
| 126 | 1 | AY723287-MY200301-SH | 0 | 0 | 0 | 0 | 0 | 0 | 1 | 0 | 0 | 0 | 0 |
| 127 | 1 | EF153678-ARK142Q-SH | 0 | 0 | 0 | 0 | 0 | 0 | 1 | 0 | 0 | 0 | 0 |
| 128 | 1 | KF830261-AG151RQ-SH | 0 | 0 | 0 | 0 | 0 | 0 | 1 | 0 | 0 | 0 | 0 |
| 129 | 15 | AF180389-A143RQ-SH | 0 | 0 | 0 | 0 | 0 | 0 | 15 | 0 | 0 | 0 | 0 |
| 130 | 1 | AJ000681-AQQ211-SH | 0 | 0 | 0 | 0 | 0 | 0 | 1 | 0 | 0 | 0 | 0 |
| 131 | 1 | AJ000679-AT137Q-SH | 0 | 0 | 0 | 0 | 0 | 0 | 1 | 0 | 0 | 0 | 0 |
| 132 | 8 | DQ677087-Gy28-SH | 0 | 0 | 0 | 0 | 0 | 0 | 8 | 0 | 0 | 0 | 0 |
| 133 | 1 | DQ677065-GD22-SH | 0 | 0 | 0 | 0 | 0 | 0 | 1 | 0 | 0 | 0 | 0 |
| 134 | 1 | DQ677055-GD8-SH | 0 | 0 | 0 | 0 | 0 | 0 | 1 | 0 | 0 | 0 | 0 |
| 135 | 1 | DQ677054-GD6-SH | 0 | 0 | 0 | 0 | 0 | 0 | 1 | 0 | 0 | 0 | 0 |
| 136 | 1 | DQ677050-DW28-SH | 0 | 0 | 0 | 0 | 0 | 0 | 1 | 0 | 0 | 0 | 0 |
| 137 | 1 | DQ677045-DW23-SH | 0 | 0 | 0 | 0 | 0 | 0 | 1 | 0 | 0 | 0 | 0 |
| 138 | 1 | DQ677043-DW21-SH | 0 | 0 | 0 | 0 | 0 | 0 | 1 | 0 | 0 | 0 | 0 |
| 139 | 1 | DQ677042-DW20-SH | 0 | 0 | 0 | 0 | 0 | 0 | 1 | 0 | 0 | 0 | 0 |
| 140 | 1 | DQ677042-DW20-SH | 0 | 0 | 0 | 0 | 0 | 0 | 1 | 0 | 0 | 0 | 0 |
| 141 | 1 | AY228473.1_Odocoileu | 0 | 0 | 0 | 0 | 0 | 0 | 0 | 1 | 0 | 0 | 0 |
| 142 | 1 | AY237542.1_Cervus_el | 0 | 0 | 0 | 0 | 0 | 0 | 0 | 0 | 1 | 0 | 0 |
| 143 | 1 | AY286008.1_Odocoileu | 0 | 0 | 0 | 0 | 0 | 0 | 0 | 0 | 0 | 1 | 0 |
| 144 | 1 | AY286007.1_Cervus_da | 0 | 0 | 0 | 0 | 0 | 0 | 0 | 0 | 0 | 0 | 1 |
| 145 | 1 | AY029774-SH | 0 | 0 | 0 | 0 | 0 | 0 | 1 | 0 | 0 | 0 | 0 |
| 146 | 1 | GQ497224-HN-6404-SH | 0 | 0 | 0 | 0 | 0 | 0 | 1 | 0 | 0 | 0 | 0 |
| 147 | 1 | AY350247-3A3Tamu-PAR | 0 | 0 | 0 | 0 | 0 | 0 | 1 | 0 | 0 | 0 | 0 |
| 148 | 2 | DQ677228-ZY126-SH | 0 | 0 | 0 | 0 | 0 | 0 | 2 | 0 | 0 | 0 | 0 |
| 149 | 1 | DQ677221-ZT29-SH | 0 | 0 | 0 | 0 | 0 | 0 | 1 | 0 | 0 | 0 | 0 |
| 150 | 1 | DQ677211-XW26-SH | 0 | 0 | 0 | 0 | 0 | 0 | 1 | 0 | 0 | 0 | 0 |
| 151 | 1 | DQ677202-XW9-SH | 0 | 0 | 0 | 0 | 0 | 0 | 1 | 0 | 0 | 0 | 0 |
| 152 | 1 | DQ677195-WD28-SH | 0 | 0 | 0 | 0 | 0 | 0 | 1 | 0 | 0 | 0 | 0 |
| 153 | 3 | DQ677154-T23-SH | 0 | 0 | 0 | 0 | 0 | 0 | 3 | 0 | 0 | 0 | 0 |
| 154 | 1 | KADSHF1 | 0 | 0 | 0 | 0 | 0 | 0 | 1 | 0 | 0 | 0 | 0 |
| 155 | 2 | KADSHF3 | 0 | 0 | 0 | 0 | 0 | 0 | 2 | 0 | 0 | 0 | 0 |
| 156 | 1 | KADSHF5 | 0 | 0 | 0 | 0 | 0 | 0 | 1 | 0 | 0 | 0 | 0 |
| 157 | 2 | KADSHM1 | 0 | 0 | 0 | 0 | 0 | 0 | 2 | 0 | 0 | 0 | 0 |
| 158 | 5 | KADSHM15 | 0 | 0 | 0 | 0 | 0 | 0 | 5 | 0 | 0 | 0 | 0 |
| 159 | 4 | KATSHF4 | 0 | 0 | 0 | 0 | 0 | 0 | 4 | 0 | 0 | 0 | 0 |
| 160 | 1 | KATSHF7 | 0 | 0 | 0 | 0 | 0 | 0 | 1 | 0 | 0 | 0 | 0 |
| 161 | 1 | KATSHM2 | 0 | 0 | 0 | 0 | 0 | 0 | 1 | 0 | 0 | 0 | 0 |
| 162 | 2 | TARSHF4 | 0 | 0 | 0 | 0 | 0 | 0 | 2 | 0 | 0 | 0 | 0 |
